# Differential Neuregulin 1 Cleavage in the Prefrontal Cortex and Hippocampus in Schizophrenia and Bipolar Disorder: Preliminary Findings

**DOI:** 10.1371/journal.pone.0036431

**Published:** 2012-05-10

**Authors:** Ketan Marballi, Dianne Cruz, Peter Thompson, Consuelo Walss-Bass

**Affiliations:** 1 University of Texas Health Science Center at San Antonio, Department of Cellular and Structural Biology, San Antonio, Texas, United States of America; 2 Southwest Brain Bank, Department of Psychiatry, University of Texas Health Science Center at San Antonio, San Antonio, Texas, United States of America; 3 Department of Psychiatry, University of Texas Health Science Center at San Antonio, San Antonio, Texas, United States of America; Alexander Flemming Biomedical Sciences Research Center, Greece

## Abstract

**Background:**

Neuregulin 1 (*NRG1*) is a key candidate susceptibility gene for both schizophrenia (SCZ) and bipolar disorder (BPD). The function of the NRG1 transmembrane proteins is regulated by cleavage. Alteration of membrane bound-NRG1 cleavage has been previously shown to be associated with behavioral impairments in mouse models lacking expression of NRG1-cleavage enzymes such as BACE1 and gamma secretase. We sought to determine whether alterations in NRG1 cleavage and associated enzymes occur in patients with SCZ and BPD.

**Methodology/Principal Findings:**

Using human postmortem brain, we evaluated protein expression of NRG1 cleavage products and enzymes that cleave at the external (BACE1, ADAM17, ADAM19) and internal (PS1-gamma secretase) sides of the cell membrane. We used three different cohorts (Controls, SCZ and BPD) and two distinct brain regions: BA9-prefrontal cortex (Controls (n = 6), SCZ (n = 6) and BPD (n = 6)) and hippocampus (Controls (n = 5), SCZ (n = 6) and BPD (n = 6)). In BA9, the ratio of the NRG1 N-terminal fragment relative to full length was significantly upregulated in the SCZ cohort (Bonferroni test, p = 0.011). ADAM17 was negatively correlated with full length NRG1 levels in the SCZ cohort (r = –0.926, p = 0.008). In the hippocampus we found significantly lower levels of a soluble 50 kDa NRG1 fragment in the two affected groups compared the control cohort (Bonferroni test, p = 0.0018). We also examined the relationship of specific symptomatology criteria with measures of NRG1 cleavage using the Bipolar Inventory of Signs and Symptoms Scale (BISS) and the Montgomery Åsberg Depression Rating Scale (MADRS). Our results showed a positive correlation between ADAM19 and psychosis (r = 0.595 p = 0.019); PS1 and mania (r = 0.535, p = 0.040); PS1 and depression (r = 0.567, p = 0.027) in BA9, and BACE1 with anxiety (r = 0.608, p = 0.03) in the hippocampus.

**Conclusion/Significance:**

Our preliminary findings suggest region-specific alterations in NRG1 cleavage in SCZ and BPD patients. These changes may be associated with specific symptoms in these psychiatric disorders.

## Introduction

Schizophrenia (SCZ) and bipolar disorder (BPD), two of the most severe psychiatric disorders, are complex disorders with both genetic and environmental underpinnings. Numerous genes have been reported to be associated with these disorders, and thought to possibly contribute to increased risk for their development. Of these, Neuregulin 1 (*NRG1*) has been shown to be linked with both SCZ [1]–[2] and BPD [3] in different populations worldwide. Over 20 different isoforms of the *NRG1* gene are generated via alternative splicing. These proteins regulate many important functions in the nervous system including myelination, glial cell migration and synapse formation. NRG1 Type III is the most abundant isoform in the brain [4]. It is synthesized as a membrane bound pro-form that is cleaved by enzymes to generate the bioactive extracellular and intracellular fragments of the protein. The extracellular fragment contains an EGF domain which interacts with ErbB tyrosine kinase receptors and activates the MAPK, PKC and AKT pathways [5]. Enzymes responsible for external cleavage include beta-site amyloid precursor protein cleaving enzyme 1 (BACE1), and a disintegrin and a-metalloproteinases (ADAM17, 19) [6]–[7]. These enzymes generate the N terminal fragment (NTF) on the external, and the C-terminal fragment (CTF) on the internal side of the membrane. Further cleavage of the intracellular CTF by gamma secretase generates the intracellular domain (ICD), which is thought to regulate gene expression [8]. Interestingly, BACE1 and Aph1BC (a component of the gamma secretase complex) knock-out mouse models, in which NRG1 cleavage is impaired, exhibit alterations in behaviors such as deficits in prepulse inhibition, cognitive impairments, alterations in social recognition and interaction [9]–[10], suggesting that imbalances in NRG1 cleavage may cause behavioral abnormalities similar to those observed in SCZ. In this study we sought to test the hypothesis that deficits in NRG1 cleavage and NRG1- cleavage enzymes may occur in SCZ in the hippocampus [11]–[14] and dorsal lateral prefrontal cortex [15]–[16]. Our laboratory previously identified a novel missense polymorphism (V>L) in the transmembrane region of NRG1 that is associated with schizophrenia [17]. This amino acid change has more recently been shown to impede gamma-secretase cleavage of the NRG1 CTF [9]. We therefore hypothesized that reduced levels of the NRG1 ICD would be found in the postmortem brain of SCZ patients. In addition, given the studies showing behavioral alterations in BACE-1 knock-out mice, we hypothesized that alterations in NRG1 external cleavage would be found in SCZ. Previous postmortem studies investigating NRG1 protein expression have shown different results, ranging from no changes [18], [19], a decrease in a 50 kDa band in BA6 in SCZ [20], and an increase in a 53 kDa band in the prefrontal cortex cytoplasmic fraction of SCZ patients [21]. Our study is novel in that we assessed levels of different NRG1 cleavage products in two distinct cellular fractions- membrane and soluble. Our reasons for doing so were two fold. First, given that NRG1 Type III has two transmembrane regions, and its cleavage products are tethered to the membrane, we anticipated that the use of the membrane fraction would facilitate detection of these products. Similarly, cleavage on the internal side would generate fragments detectable in the soluble fraction. Second, the field of NRG1 research is plagued with a dearth of non-specific antibodies, and probing each fraction individually eliminated some of the non specificity. In addition to NRG1 levels, we investigated levels of the main enzymes that are involved in cleavage of NRG1, to test the hypothesis that alterations in enzyme levels would correlate with changes seen in NRG1 cleavage. We used three groups (control, SCZ and BPD patients) and two different brain regions (prefrontal cortex and hippocampus). Based on the diverse functionality of these two regions, we hypothesized that we would observe region specific changes in NRG1 cleavage. Additionally, because of the interest in determining intermediate phenotypes (endophenotypes) and assessing symptomatology that cuts across diagnostic criteria, we examined whether specific symptom clusters were associated with the biological measures being tested. To do so, we analyzed symptoms experienced in the last week of life using scales of symptom severity such as the MINI [22] Bipolar Inventory of Signs and Symptoms Scale (BISS) [23] and the Montgomery Åsberg Depression Rating Scale (MADRS) [24]. These studies will shed light as to the role of NRG1 in development of SCZ and BPD.

## Results

### Sample Demographics and Variables

For the BA9 comparisons, no significant differences were found between the demographic variables pH, PMI, brain weight, race and age (Tables S1 and S2). Given that the control group consisted solely of males, to determine if gender had an effect on protein levels, we combined the SCZ and BPD groups and found no significant differences on the effect of gender (T test, p = 0.07) on protein levels. For the hippocampal area studies, age was significantly different between the 3 groups, (F (2, 14)  = 6.040, p = 0.013) (Bonferroni post hoc test showed the BPD cohort to be significantly different from controls, p = 0.011). Pearson’s correlation analyses were carried out between the different demographic criteria (age, race, sex, pH, PMI, brain weight) and all the different proteins assayed. In BA9, pH was found to correlate with ADAM17 (r = –0.523, p = 0.026); NRG1 NTF (r = 0.530, p = 0.024) and NRG1 FL (r = 0.487, p = 0.04); brain weight was shown to correlate with NRG1 FL (r = 0.473, p = 0.047). In the hippocampus, sex correlated with BACE1 levels (r = 0.563, p = 0.018) and race correlated with NTF (r = –0.541, p = 0.025); brain weight was shown to negatively correlate with BACE1 levels (r = –0.527, p = 0.030). Based on these results, ANCOVA was used to determine differences between groups using pH as a covariate for ADAM17, NRG1 NTF and FL; brain weight as a covariate for NRG1 FL in BA9; sex as a covariate for BACE 1, and race as a covariate for NTF in the hippocampus. ADAM17 was found to be significantly different between groups when using pH as a covariate (F (2, 15) = 7.166, p = 0.007). None of the other ANCOVAs were significant. Thereafter ANOVA was used to determine differences between groups for the remaining proteins (that did not show significant Pearson’s correlations with any of the demographic variables), followed by post hoc analyses using Bonferroni test.

Four different classes of medication were used by the disease cohort, namely-antipsychotics, antidepressants, benzodiazepines and mood stabilizers (Table S3). Since the cohort sizes were too small to determine the effects of each of the different classes of drugs, we stratified the disease cohort into two groups, those that were on medication and those that were not on any medication. No effects of medication on protein levels were observed in the combined SCZ and BPD group in BA9 (T test, p = 0.376) or hippocampus (T test, p = 0.155).

### NRG1 Protein Expression in Different Brain Regions in SCZ and BPD

For detection of the NRG1 full length and post-external cleavage fragments, we used an antibody against NRG1 type III (antibody ab5551), that recognizes the Full length (FL) (expected size-130 kDa) and the N-terminal fragment (NTF) (expected size -76 kDa) [25]. Given that NRG1 type III has two transmembrane domains, the NTF remains attached to the membrane after initial cleavage by external cleavage enzymes [25]. For detection of the soluble internal cleavage product, we used an antibody against the cytoplasmic tail (sc348 antibody), which is cleaved by gamma secretase to generate the intracellular domain (ICD) in the soluble fraction. The expected size of this band is 53 kDa [20].

**Figure 1:**
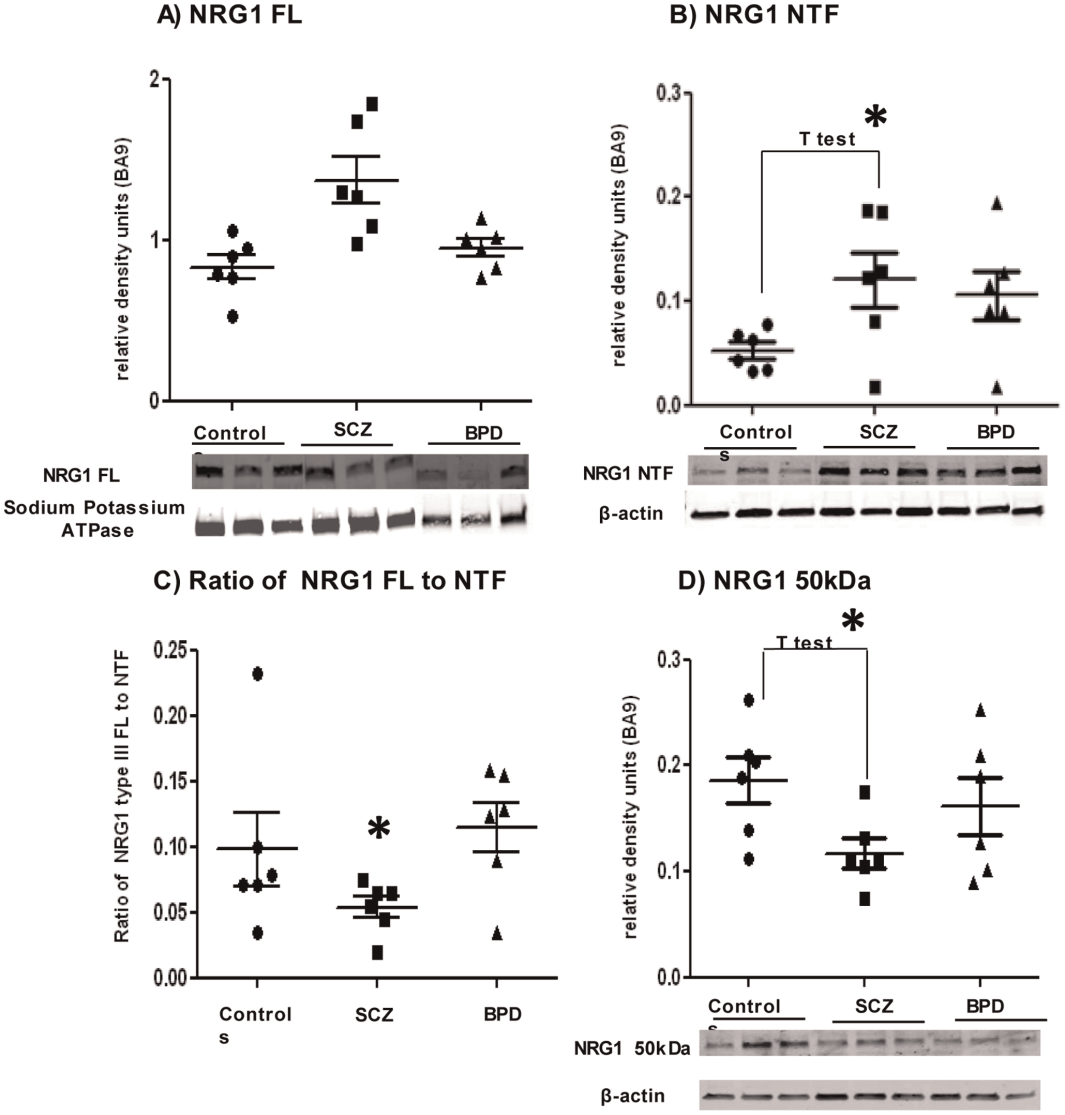
Levels of Neuregulin 1 (NRG1) in Brodmann’s area 9 (BA9). Representative western blots for each protein, with three samples for each group, are shown. Densitometric analyses for all samples are shown above the western blot images. Values represent relative levels of protein bands, normalized to sodium potassium ATPase for Full Length (FL), or beta actin for N-terminal fragment (NTF). 1A) NRG1 FL; 1B) NRG1 NTF; 1C) Ratio of NTF/FL; 1D) NRG1 50 kDa. Controls (•), schizophrenia -SCZ (▪), and bipolar disorder -BPD (▴). 1C shows the NTF/FL to be significantly higher, by Bonferroni post-hoc test, in the SCZ cohort compared to controls (p = 0.011) and BPD (p = 0.027). 1D shows NRG1 50 kDa levels are significantly lower in SCZ compared to controls (T test, p = 0.023). Asterisk indicates statistical significance. Graphs are plotted as mean ± standard error.

In BA9, the FL 130 kDa and NTF 70 kDa protein bands were detected in the membrane fraction. ANOVA showed no significant differences in the levels of these bands between the three different patient groups (Fig.1A). Based on the studies showing BACE1 and gamma secretase knockout mice with SCZ-like behavioral abnormalities, we had an *apriori* hypothesis that the cleavage impairments would be specific to SCZ. Therefore, T tests were performed and significantly higher levels of the NTF in SCZ compared to controls (p = 0.0335) (Fig.1B), but not in BPD compared to controls, were found. Furthermore, the ratio of the NTF to FL was significantly higher in the SCZ cohort by ANOVA (F (2, 15) = 6.997, p = 0.007) (Fig.1C). Bonferroni post hoc test showed the SCZ group to be significantly different from controls (p = 0.011) and BPD (p = 0.027), providing additional evidence of altered NRG1 extracellular processing in SCZ (Fig.1). In the soluble fraction, no significant differences between groups were found in the levels of the 50 kDa NRG1 band by ANOVA. However, levels were shown to be significantly lower in SCZ compared to controls by T test. (p = 0.0239) (Fig. 1D). This supported our *apriori* hypothesis that reduced levels of the NRG1 intracellular fragment would be found in SCZ.

**Figure 2:**
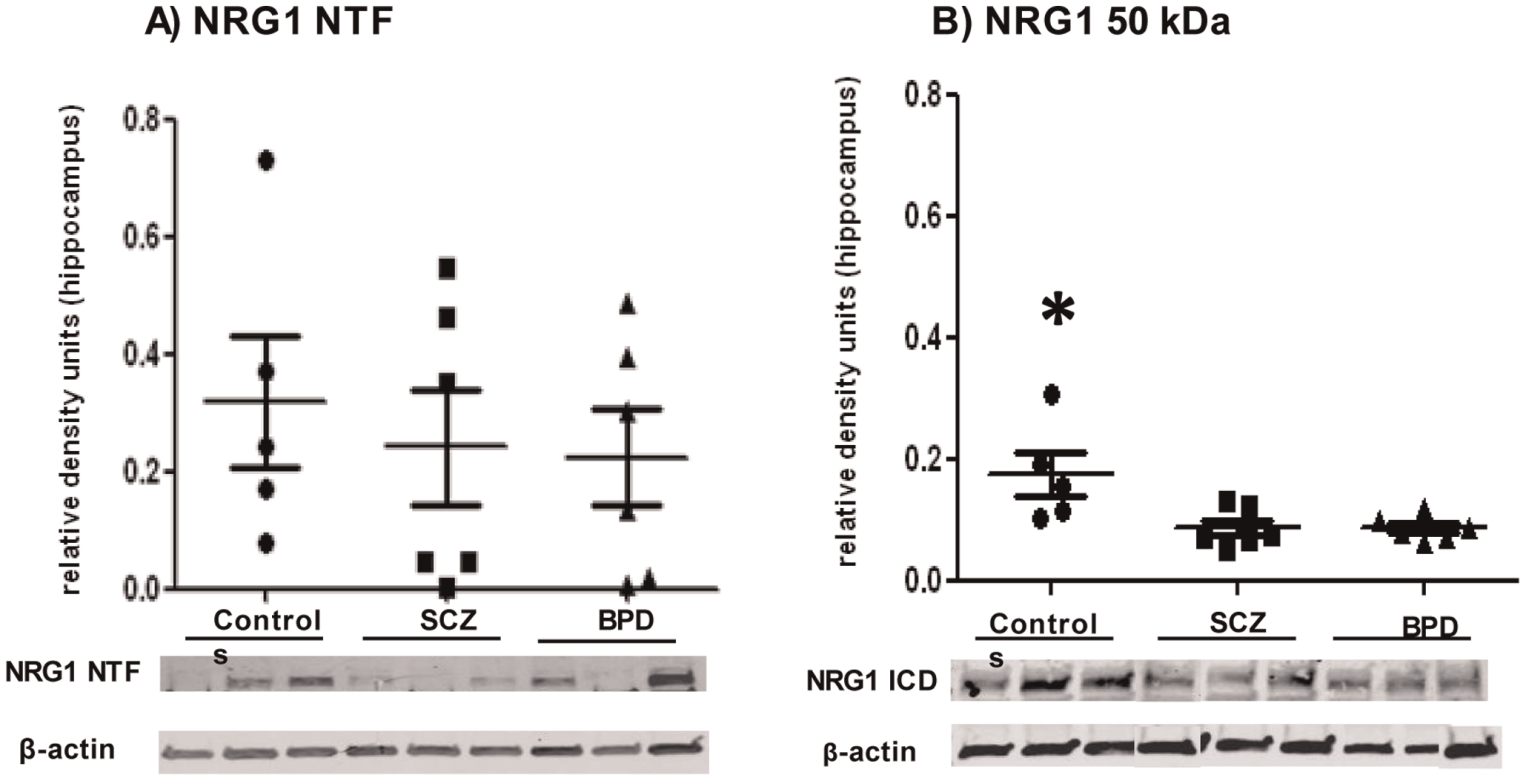
Levels of Neuregulin 1 (NRG1) in the hippocampus. Values represent relative levels of NRG1, normalized to beta actin. 2A) NRG1 NTF; 2B) NRG1 50 kDa. Controls (•), schizophrenia -SCZ (▪), and bipolar disorder -BPD (▴). Bonferroni post hoc analyses show controls have significantly higher levels of the soluble NRG1 50 kDa band compared to SCZ (p = 0.018) and BPD (p = 0.022). Asterisk indicates statistical significance. Graphs are plotted as mean ± standard error.

In the hippocampus, there were no significant differences between groups in the levels of the NTF 70 kDa in the membrane fraction (Fig. 2A). In the soluble fraction, significantly lower levels of the NRG1 50 kDa protein band were observed in the affected cohorts compared to controls (Fig. 2) ANOVA(F(2, 15) = 6.596, p = 0.010). Bonferroni post hoc test showed controls significantly different from SCZ (p = 0.018) and BPD (p = 0.022) (Fig. 2).

### Expression of NRG1 Cleavage Enzymes and Correlation with NRG1 Cleavage Products

**Figure 3:**
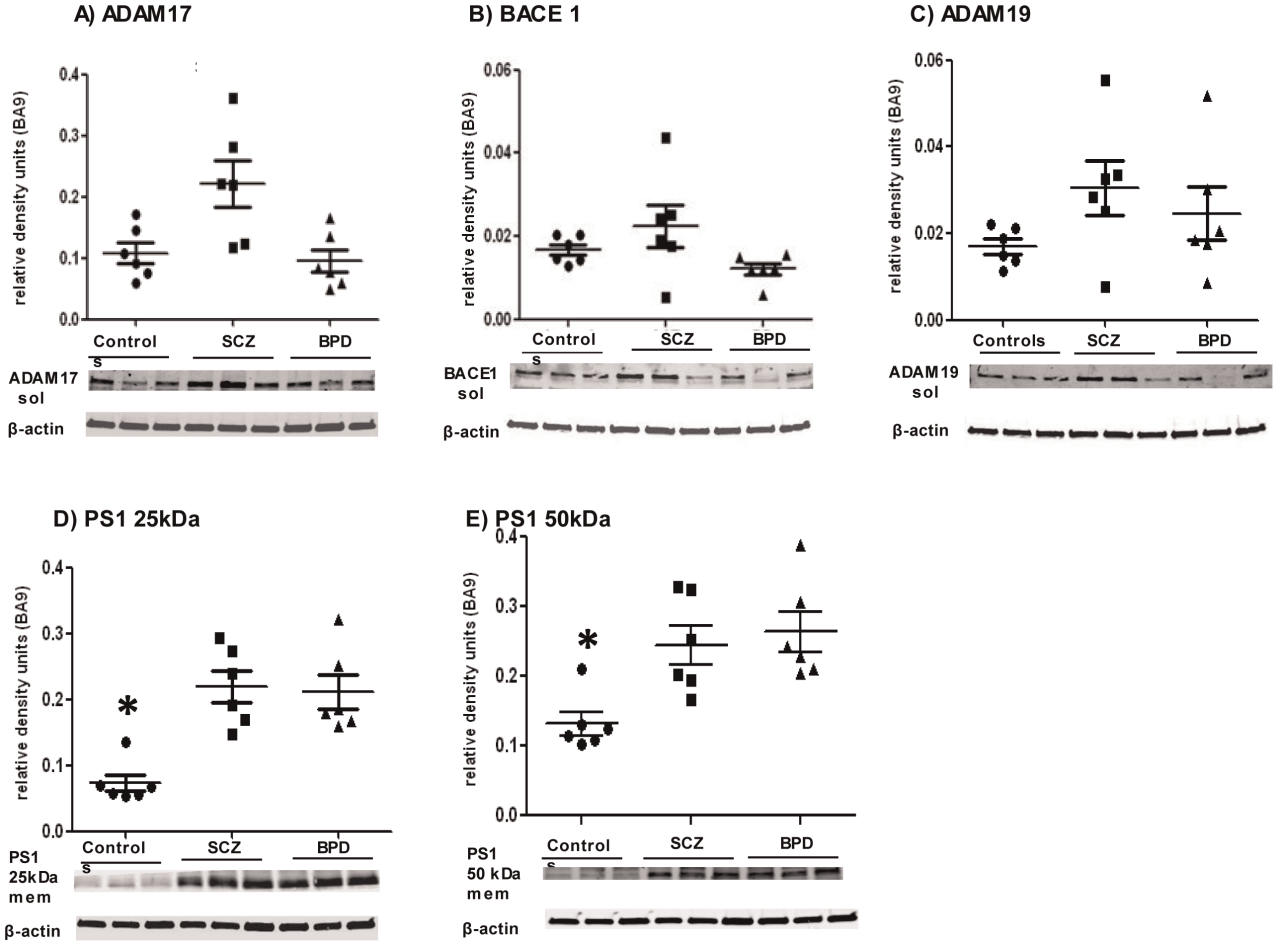
Levels of Neuregulin 1 (NRG1) cleavage enzymes Presenilin 1 (PS1) and ADAM17 in Brodmann’s Area 9 (BA9). Values represent relative levels of protein bands, normalized to beta actin. 3A) ADAM17; 3B) BACE1; 3C) ADAM19; 3D) PS1 50 kDa; and 3E) PS1 25 kDa. Controls (•), schizophrenia -SCZ (▪) and bipolar disorder -BPD (▴). 1A) shows levels of ADAM17 to be significantly different between cohorts by ANCOVA [F (2, 15) = 7.166, p = 0.007]. 3D) and E) show PS-1 25 and 50 kDa levels are significantly lower in SCZ by Bonferroni post hoc analyses, compared to controls (p = 0.001) and BPD (p<0.001). Asterisk indicates statistical significance. Graphs are plotted as mean ± standard error.

We measured levels of different known NRG1- cleavage enzymes, namely ADAM17, ADAM19 and BACE1 that are responsible for external cleavage, and Presenilin 1 (PS1) - the catalytic subunit of gamma secretase, which is the enzyme that cleaves NRG1 on the internal side. Levels of the external cleavage enzymes were assessed in the soluble fractions, as these proteins are known to be localized in both the membrane and cytosol [26], [27] and we had more protein available from the soluble fraction. Pilot experiments showed that the expression patterns for these enzymes were the same in both the membrane and soluble fractions (data not shown). ANCOVA analyses showed that ADAM17 was significantly different between groups after taking pH as a covariate in BA9 (F (2, 15) = 7.166, p = 0.007) (Fig. 3A). BACE1 and ADAM19 (Fig. 3B, 3C) were not significantly different between groups in BA9. PS1 levels were measured in the membrane fraction, as it has been found to be localized in the plasma membrane [28]. In BA9, PS1 levels were found to be significantly different in the control group compared to the affected cohorts (Fig. 3D, E). Two distinct protein bands were observed for PS1 (50 and 25 kDa). For the 50 kDa band, ANOVA (F (2, 15) = 12.551, p = 0.001) with post-hoc Bonferroni test showed significantly higher levels in both SCZ (p = 0.003) and BPD (p = 0.001) (Fig. 3E). A similar observation was made with the 25 kDa band, where ANOVA (F (2, 15) = 26.824, p<0.001) and post hoc Bonferroni tests showed higher levels in SCZ and BPD compared to controls (Fig. 3D). No significant differences were observed in BACE1 or ADAM19 protein levels in BA9. No significant differences between the groups were seen in the hippocampal region in the levels of any of the cleavage enzymes (not shown).

**Figure 4:**
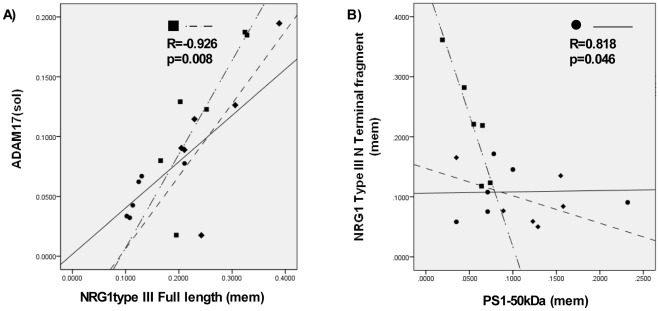
Pearson’s correlation analyses between levels of Neuregulin 1 (NRG1) and NRG1 cleavage enzymes in Brodmann’s area 9 (BA9). A) ADAM17 and NRG1 full length; B) NRG1 NTF and PS1-50 kDa. Controls (•, —); SCZ (▪, •—•); BPD (♦, –-). Inset shows R and p values for the statistically significant cohort (represented by symbol) for each plot.

To ascertain if there was a relationship between the levels of cleavage enzymes and NRG1 protein levels, correlation analyses were carried out between the different NRG1 protein products and cleavage enzymes respectively. Correlations were considered significant when p<0.004, after correcting for multiple testing (12 correlations; 4 enzymes and 3 NRG1 protein products. In BA9, levels of full length NRG1 protein were negatively correlated with levels of ADAM17 (r = –0.547, p = 0.019), with the SCZ cohort showing a negative correlation (r = –0.926, p = 0.008) that was not seen in the other two groups (Fig. 4A). Levels of the 70 kDa NRG1 NTF band were positively correlated with levels of the PS1-50 kDa in BA9, in the control cohort only (r = 0.818, p = 0.046) (Fig. 4B), however this was not significant after correcting for multiple testing. No correlations between NRG1 protein levels and enzyme levels were observed in the hippocampus.

### Correlations Between Symptom Clusters and Biological Measures

**Figure 5:**
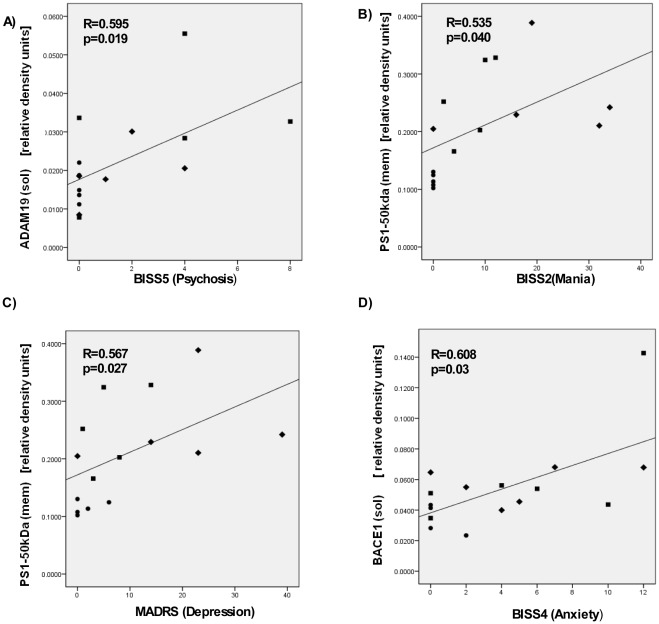
Pearson’s correlation analyses between levels of NRG1 cleavage enzymes and symptomatology criteria. A, B, C) BA9; D) hippocampus; A) ADAM19 and BISS5 (Psychosis); B) Presenilin-1 (PS1)-50 kDa and BISS2 (Mania); C) PS1-50 kDa and MADRS (Depression); D) BACE1 and BISS4 (Anxiety). Controls (•), SCZ (▪) and BPD (▴). R and p values were calculated based on symptomalogy score and protein expression, regardless of DSM diagnosis.

To determine if the biological measures were associated with specific symptomatology, we performed Pearson’s correlations using the BISS and MADRS scales of symptom severity. We used total severity scores for each scale and individual symptom factor scores for the BISS. All samples, controls and patients, were analyzed together based on symptom scores, since some of the controls had basal levels of scores for these instruments. In BA9 we found a positive correlation between ADAM19 levels and the BISS psychosis factor (r = 0.595 p = 0.019) (Fig. 5A). PS1-50 kDa levels showed a positive correlation with the mania factor (r = 0.535, p = 0.040) (Fig. 5B). Depression scores measured by MADRS also correlated positively with the PS1-50 kDa band (r = 0.567, p = 0.027) (Fig. 5C). In the hippocampal region, BACE1 showed a positive correlation with the BISS anxiety factor (r = 0.608, p = 0.03) (Fig. 5D. Correlations are considered significant when p<0.001, after correcting for multiple testing (49 correlations; 4 enzymes and 3 NRG1 protein products (total 7) and 7 symptom measures). Importantly, when only patient samples (excluding controls) were analyzed, the correlations were no longer observed.

## Discussion

In this exploratory study we aimed to determine whether alterations in NRG1 protein cleavage and in expression of NRG1 cleavage enzymes are present in SCZ and BPD, and if so, is there anatomical specificity. As stated by Boer et al, previous studies have shown inconsistent results with regards to levels of different NRG1 cleavage products [19]. Our approach was to use membrane and soluble brain fractions to estimate levels of membrane bound (full length, N-terminal fragment) and soluble (intracellular domain) NRG1 products, and to investigate different cleavage enzymes responsible for internal and external cleavage. We also used a novel approach to identify correlations between specific symptomatology and our biological findings, to identify alternate phenotypes to the DSM (Diagnostic and Statistical Manual, American Psychiatric Association) (DSM-IV-TR, 2000) [29]. Using human post mortem brain tissue, we examined two different brain regions, BA9 and the hippocampus, which have been implicated in SCZ and BPD. BA9 of the prefrontal cortex has been associated with deficits in cognitive dysfunction in SCZ [30] and BPD [31]. The hippocampus regulates the process of memory formation, which is known to be affected in both SCZ and BPD patients [32]. Additionally, the role of NRG1 in the hippocampus has been widely investigated [33]–[35].

Our results showed no significant differences in full length NRG1 tethered to the membrane, but we did observe changes when we compared the ratio of the external cleavage product to the full length protein in BA9. We observed significantly higher levels of the NTF/FL band ratios in the SCZ cohort, suggesting that extracellular NRG1 processing is altered in SCZ. In support of this, we observed that levels of ADAM17 were correlated negatively with levels of full length protein in the SCZ cohort compared to the control and BPD cohorts. Elevated levels of ADAM17 could give rise to the higher levels of NTF (relative to FL) in the SCZ cohort (also shown by significantly higher levels of NTF in SCZ vs. controls by T test). However, we did not observe a positive correlation between ADAM17 levels and NTF levels. External cleavage is known to be performed by enzymes such as BACE1, ADAM19 and ADAM17, which cut on distinct sites of the protein. However, the sequence in which these events take place is not fully characterized. It is possible that cleavage of the FL NRG1 by ADAM 17 occurs early on, while additional enzymes or factors regulate formation of the final NTF fragment. For example, cytokines such as IL-6 have been shown to regulate cleavage of FL NRG1 [36]. This may explain why correlation of ADAM17 was observed with reduced levels of FL, but not with increased levels of NTF. Increased extracellular cleavage of NRG1 could potentially lead to aberrant activation of ErbB receptors and signaling pathways such as the ERK, PKC and AKT pathways. Increased activation of the ErbB4 receptor induced by NRG1 was previously reported in a SCZ postmortem brain study [37]. Also, increased activation of ERK2 has been reported in SCZ patients [38]. The differences in NTF/FL and ADAM17 levels were not observed in the hippocampus in SCZ patients, or in BPD in either region. There is additional evidence in the literature of region specific changes in NRG1 in schizophrenia. Gene expression of NRG1 isoforms has been observed to be different in the prefrontal cortex and hippocampus [39]. In a chemically induced- rat model of schizophrenia, it was shown that NRG1-beta protein levels were significantly higher in the CA1, CA3 and dentate gyrus in the hippocampus, but only slight increases were seen in the prefrontal cortex compared to the control group [40].

We observed significantly lower levels of the NRG1 50 kDa band by ANOVA in the hippocampus in both affected cohorts, and T tests showed significantly lower levels of this band in BA9 in the SCZ cohort compared to controls. Although in a functionally different brain region, this is consistent with previous results obtained by Barakat *et al*, where lower levels of a NRG1 50 kDa band were observed in the agranular frontal cortex region (BA6) in SCZ patients [21]. We speculate that this 50 kDa band could be the intracellular domain (ICD); however due to the lack of specific antibodies against the ICD we cannot definitively ascertain the identity of this band. Another study by Chong et al reported that levels of a NRG1 53 kDa band (possibly the ICD) were increased in the prefrontal cortex cytoplasmic fraction in SCZ patients [19]. This study did not specify the specific region within the PFC, so it is possible that the observed differences in NRG1 levels compared to our study may be due to different brain regions being used. The results from these studies suggest that even within the frontal cortex, there may be differences in NRG1 cleavage based on sub-anatomical regions. Our laboratory previously reported the finding of a NRG1 V>L transmembrane polymorphism that is associated with SCZ [17]. This variant has now been found to inhibit gamma secretase-induced release of the NRG1 ICD [9], [41], and has also been shown to cause reduced cortical neuron dendrite formation *in vitro* [41]. Inhibition of gamma secretase cleavage of NRG1 has been shown to induce SCZ-like symptoms in an animal model [9]. Our finding of decreased levels of the NRG1 50 kDa band (possibly the ICD) in the SCZ cohort is consistent with our *apriori* hypothesis that reduced levels of NRG1 intracellular cleavage would be observed in SCZ. However, importantly, although we did not observe differences in levels of the 50 kDa band in BA9 in the BPD cohort, we did observe differences in this band in the hippocampus in BPD. The small sample size we used, giving the study low power, could be the reason for the non-significant results for the BPD cohort in BA9. Alternatively, there could in fact be region-specific differences in the role of NRG1 in SCZ and BPD. Additional studies using larger cohorts are necessary to determine whether this is true. We also hypothesized that changes in enzyme levels would correlate with changes in NRG1 cleavage. However, we observed higher levels of PS1 in the SCZ and BPD cohorts in BA9, as opposed to the lower levels to be expected given the lower levels of the soluble 50 kDa band (ICD). It is possible that the increase in PS1 levels is due to increased extracellular cleavage, which generates the protein product to be further cleaved by gamma secretase [25]. This may also be an explanation of why levels of PS1 did not correlate with the 50 kDa band levels, but were positively correlated with the NTF band. An alternate explanation could be that lower levels of the putative ICD were observed in the affected cohorts not because of differences in levels of enzyme expression, but due to alterations in activity of the gamma secretase enzyme (not tested), or due to inherent NRG1 mutations, such as the V>L variant, which may impede cleavage. This may also be the reason why we did not observe changes in PS1 expression in the hippocampus across the different cohorts, although differences in ICD levels were observed. Further studies are necessary to determine the specific cause of the observed alterations in levels of the NRG1 50 kDa band.

In summary, we have obtained preliminary results pointing towards region-specific and disease-specific alterations in NRG1 cleavage in postmortem brain. We hypothesize that the region specific differences we see may allude to the role of NRG1 in different brain regions [38]–[39]. This may lead to differences in pathology and symptomatology occurring in SCZ and BPD. Some of our significant findings of altered NRG1 cleavage and enzyme levels were observed in SCZ and not BPD, such as levels of ADAM 17 and NTF being specifically higher in SCZ, compared to BPD and controls. However, other findings, such as increased levels of PS1, were seen for both SCZ and BPD in BA9. Interestingly, when we analyzed the data using specific symptomatology criteria, we found positive correlations between levels of PS1 and BISS mania scores, and PS1 and MADRS total scores in BA9. This suggests that alterations in this enzyme may be associated with specific symptoms, which are present in both BPD and SCZ, and argue in favor of the use of alternative phenotypes when investigating the biological mechanisms of psychiatric disorders. However, in all cases, our results may be confounded due to the small sample sizes used, leading to low power, and therefore the non-significant results may be false negatives. The small sample size is also the reason for the large amount of variability observed in some of the measures. Therefore, we emphasize that the present results, while important, are preliminary and must be interpreted with caution. These results must be replicated using additional and larger cohorts.

In summary, NRG1 cleavage is a complex process that appears to be regulated in an enzyme –dependent, region-dependent manner, and investigating these pathways will help to gain a better understanding of the role of NRG1 in psychiatric pathology. While the small sample sizes we used is a great limitation, this study is novel in that we examined levels of NRG1 cleavage enzymes and levels of NRG1 protein in two psychiatric disorders and two distinct brain regions. We encourage additional studies with larger cohorts to validate these results. In addition, we hope our exploratory studies with regards to correlation of enzyme expression with specific symptoms encourages other researchers to adopt a more holistic approach of utilizing symptomatology as a useful adjuvant for studying the molecular mechanisms of psychiatric disorders.

## Materials and Methods

### Postmortem Brain Samples

Postmortem brain tissue was obtained from the Southwest Brain Bank, Department of Psychiatry UTHSCSA. Tissue collection is under the auspices of the Anatomical Board of the State of Texas, and all interactions involving living subjects are regulated by the UTHSCSA Institutional Review Board. Next of Kin interviews were conducted by a trained clinician using the following instruments: Mini International Neuropsychiatric Interview (MINI) [21], Montgomery Åsberg Depression Rating Scale (MADRS) [22] and the Bipolar Inventory of Signs and Symptoms Scale (BISS) [23]. The BISS has 5 factors: depression, mania, irritability, anxiety, and psychosis [42]. These instruments assess symptoms experienced by the deceased in the last week of life. Consensus diagnosis for all subjects was obtained by reviewing medical records, toxicology reports and interview data according to DSM-IV criteria by an independent panel of at least three trained clinicians. Brain tissue was evaluated by a neuropathologist and was found to be free of any confounding neuropathology. Whole brains were dissected by cutting the brain coronally into 1 cm thick sections, digitally photographed, immediately frozen and stored in a –80°C freezer. From the frozen blocks, dissections of prefrontal cortex Brodmann’s area 9 (BA9) from the right hemisphere were made from the superior frontal gyrus, approximately 1.5 cm rostral to the genu of the corpus callosum. For the hippocampal region, tissues were sectioned using a cryostat and the rostral hippocampus region was isolated. For the BA9 region a cohort of controls (no DSM-IV diagnosis) (n = 6), SCZ (n = 6) and BPD (n = 6) patients was used. Of these brain samples, hippocampal tissue was only available for n = 4 controls, n = 5 SCZ and n = 5 BPD patients. Therefore additional samples were added to each group such that for the hippocampal region, a final cohort of controls (n = 5), SCZ (n = 6) and BPD (n = 6) patients was used. See Tables S1 and S2 for sample demographics.

### Preparation of Membrane and Soluble Extracts

Protein samples were prepared using the Proteoextract Native membrane protein extraction kit (EMD Biosciences, Gibbstown, New Jersey) using 50 mg of frozen tissue and homogenization was carried out using a Polytron PT-2100 bench top homogenizer (Capitol scientific Inc. Austin, Texas) at 19000 rpm for 5 seconds. Using a combination of different extraction buffers provided with the kit, membrane and soluble fractions were collected, aliquoted, and stored in –80°C. Protein concentrations were determined using a microplate version of Bradford’s assay (Pierce, Thermo scientific, Rockford, Illinois). All proteins were normalized for equal amounts of total protein and prior to gel electrophoresis, Laemmli buffer was added and samples were denatured at 95°C for 5 minutes. The quality of the membrane and soluble fractions was verified using antibodies against Sodium Potassium ATPase (ab7671) from Abcam, San Francisco California (specific for membrane) and ERK (sc-93) from Santa Cruz Biotechnology, Santa Cruz California (specific for soluble fraction). No significant cross contamination was observed between fractions (data not shown).

### Western Blot Analyses

Equal amounts of protein from the membrane (BA9 17 µg and Hippocampus 26 µg) or soluble fractions (BA9 8 µg and Hippocampus 17 µg) were loaded for each sample. Based on standard curves, all the proteins assayed were within the linear range of detection - Fig. S1. Proteins were separated on 4–20% SDS polyacrylamide gels (Bio-Rad, Hercules,California) and transferred overnight at 30 mV to Immobilon FL PVDF membranes (Millipore, Billerica, Massachusetts). This was followed by incubation with Odyssey blocking buffer (LI-COR Biosciences, Lincoln, Nebraska) for 1 hour at room temperature and then overnight incubation with primary antibodies : ab104800 (ADAM19), ab39162 (ADAM17), ab23796 (BACE1) and ab71181(PRESENILIN1) from Abcam; NRG1 type III antibody (AB5551) from Millipore, Billerica, Massachusetts; NRG1 C-terminal antibody (sc-348) from Santa Cruz Biotechnology; A1978 (Beta Actin antibody) from Sigma Aldrich, St. Lewis, Missouri; ab 7671 (Sodium Potassium ATPase) from Abcam was used as a loading control for our studies with full length NRG1. For all other measurements, based on previous studies, beta actin was used as loading control for both the membrane and soluble fractions [43]. After overnight incubation, three washes were given in phosphate buffered saline with 0.1%Tween 20 (PBS -tween) (10 minutes each) followed by incubation with fluorescently labeled secondary antibodies (IR dye 800 Goat anti-Mouse IgG (H + L) and IR dye 680 Goat anti-Rabbit IgG (H + L), from LI-COR Biosciences. This was followed by a second set of washes in PBS-tween and images were scanned on an Odyssey imager and analyzed using the Odyssey software (LI-COR Biosciences) by normalizing densities of the protein of interest to the loading control. All proteins were assayed at least twice to ensure consistency of results. Pictures of the entire blots with molecular markers are shown in Fig. S2.

### Statistical Analyses

Densitometry data from western blots for BA9 and hippocampal regions were analyzed using SPSS 18 software (IBM Armonk, New York). Data were log transformed and checked for normal distribution using the Kolmogorov-Smirnov and Shapiro-Wilk tests for homogeneity of variance and comparisons were made with either ANCOVA or ANOVA followed by the Bonferroni post hoc test and Pearson’s correlation analyses. Findings where p<.05 are considered significant for ANCOVA and ANOVA. For the NRG1 protein vs cleavage enzyme correlations, a total of 12 correlations (4 enzymes and 3 NRG1 protein products) were carried out, and therefore a p value of 0.05/12 or 0.0041 was considered significant. For correlations with symptomatology, a total of seven symptom measures were tested. Therefore a p value of 0.05/49 or 0.001 was used as a cutoff.

## Supporting Information

Figure S1 Standard curves for proteins assayed. Standard curves were generated for NRG1 FL, NRG1 NTF, PS1-50 kDa, ADAM17, NRG1 50 kDa, BACE1 and ADAM19. Range of protein loaded was 2–50 µg for membrane fraction, and 2–40 µg for soluble fraction.
(TIF)

Figure S2 Gel blots of significant data with molecular markers. Representative western blots for each protein assayed, with three samples for each group are shown. Each blot is shown with the corresponding beta actin loading pattern. NRG1 FL, NRG1 NTF, PS1-50 and 25 kDa blots are from BA9 membrane (mem) fraction. ADAM17 and NRG1 50 kDa blots are from BA9 soluble (sol) fraction. NRG1 50 kDa is from the hippocampus soluble (sol) fraction.
(TIF)

Table S1 Sample Demographics for Brodmann’s area 9 (BA9). Table S1 represents demographic information for different variables from BA9 region for the three different groups: Controls (N = 6), Schizophrenia (N = 6) and Bipolar Disorder (N = 6). This includes Age, Sex, Race, PMI (post mortem interval) in hours (hrs), Brain weight in grams (gms), pH, BISS (Bipolar Inventory of Signs and Symptoms Scale) and, MADRS (Montgomery Åsberg Depression Rating Scale). Total BISS score is cumulative of BISS1-5 (1 =  depression, 2 =  mania, 3 =  irritability, 4 =  anxiety, 5 =  psychosis). * BISS and MADRS data was not available for one sample in each group.
(DOCX)

Table S2 Sample Demographics for Hippocampus. Table S2 represents demographic information for different variables from hippocampal region for the three different groups: Controls (N = 5), Schizophrenia (N = 6) and Bipolar Disorder (N = 6). This includes Age, Sex, Race, PMI (post mortem interval) in hours (hrs), Brain weight in grams (gms), pH, BISS (Bipolar Inventory of Signs and Symptoms Scale) and, MADRS (Montgomery Åsberg Depression Rating Scale). Total BISS score is cumulative of BISS1-5 (1 =  depression, 2 =  mania, 3 =  irritability, 4 =  anxiety, 5 =  psychosis). * BISS data was not available for one sample from the control group. ** shows the bipolar cohort to be significantly different in age from the control cohort (p = 0.004).
(DOCX)

Table S3 Sample medication data. Table S3 represents medication data for different variables from all the subjects used for our study. Variables shown include sample ID, Group (Control, schizophrenia (SCZ) or bipolar disorder (BPD)); Tissue availability (Brodmann’s area 9 (BA9) and/or hippocampus) and the different classes of medications that the subjects were on, including Antidepressant, Benzodiazapine, Neuroleptics and Mood stabilizer denoted by Y-Yes or N-No.
(DOCX)
