# Supplementary figures and images for: Differential Neuregulin 1 Cleavage in the Prefrontal Cortex and Hippocampus in Schizophrenia and Bipolar Disorder: Preliminary Findings
Source: PLoS One. 2012 May 10;7(5):e36431. doi: 10.1371/journal.pone.0036431 (PMC3349664; doi:10.1371/journal.pone.0036431)

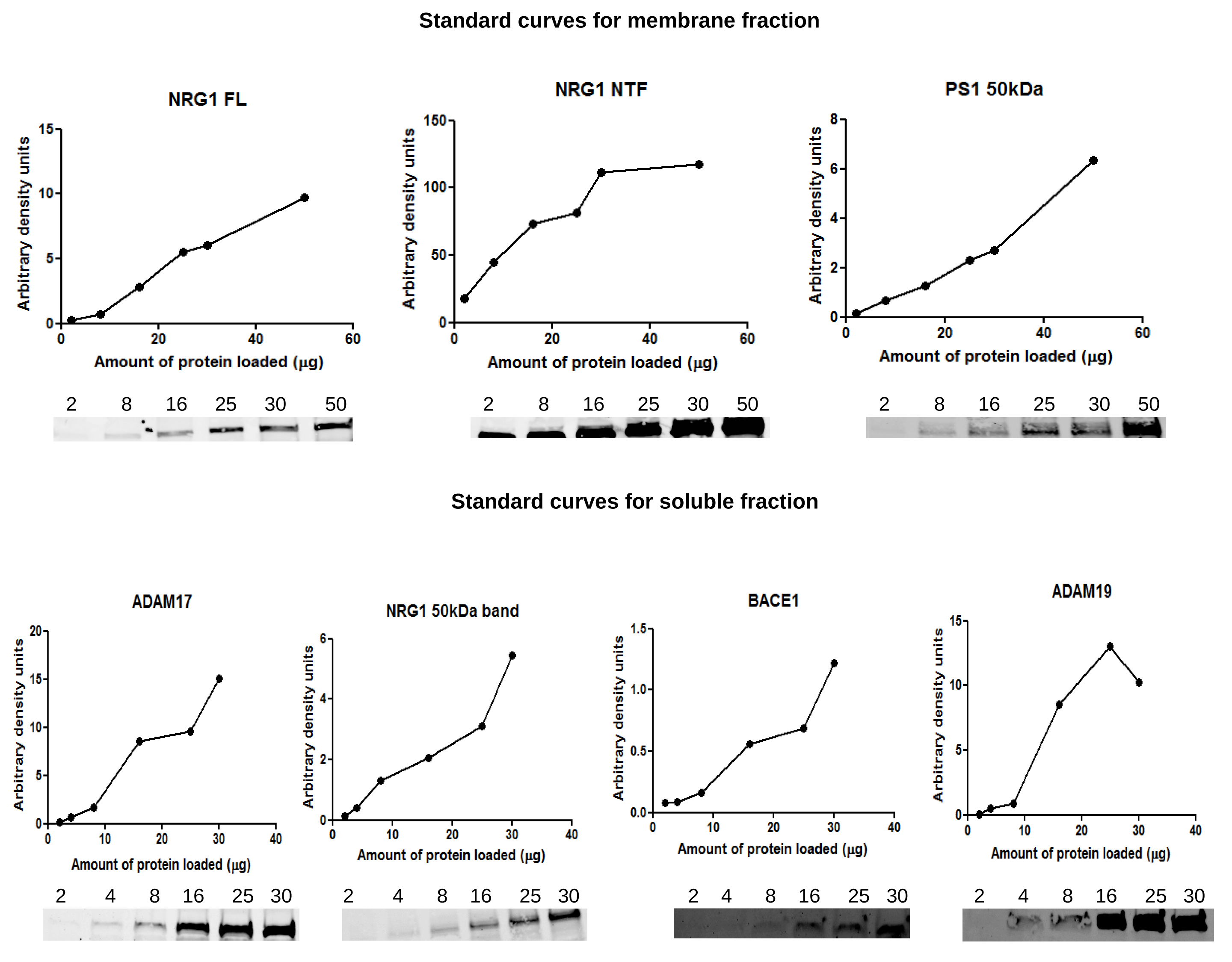

Supplement: Figure S1 — Standard curves were generated for NRG1 FL, NRG1 NTF, PS1-50 kDa, ADAM17, NRG1 50 kDa, BACE1 and ADAM19. Range of protein loaded was 2–50 µg for membrane fraction, and 2–40 µg for soluble fraction. (TIF) [file pone.0036431.s001.tif]

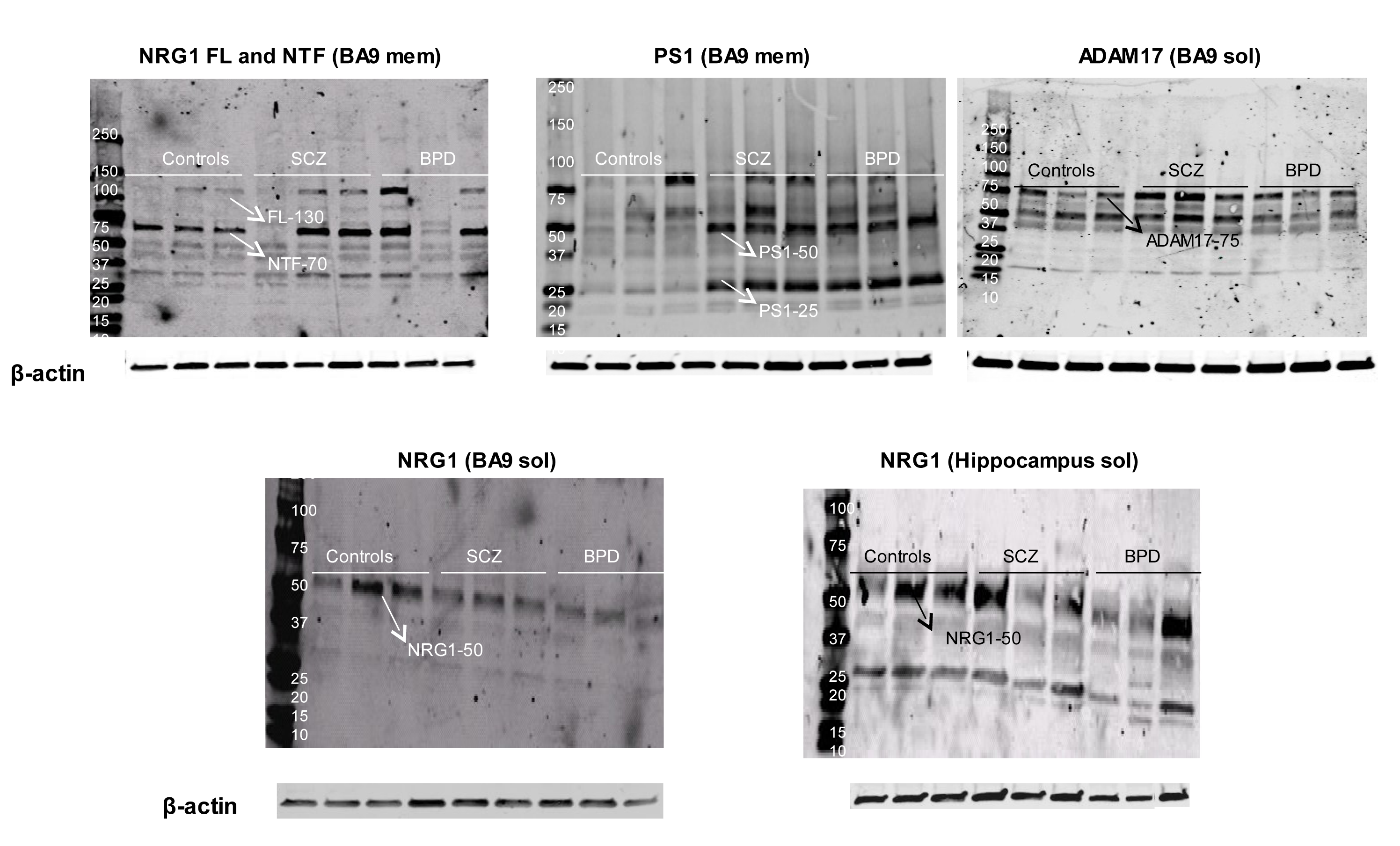

Supplement: Figure S2 — Representative western blots for each protein assayed, with three samples for each group are shown. Each blot is shown with the corresponding beta actin loading pattern. NRG1 FL, NRG1 NTF, PS1-50 and 25 kDa blots are from BA9 membrane (mem) fraction. ADAM17 and NRG1 50 kDa blots are from BA9 soluble (sol) fraction. NRG1 50 kDa is from the hippocampus soluble (sol) fraction. (TIF) [file pone.0036431.s002.tif]
