# Supplementary material for: Differential Neuregulin 1 Cleavage in the Prefrontal Cortex and Hippocampus in Schizophrenia and Bipolar Disorder: Preliminary Findings
Source: PLoS One. 2012 May 10;7(5):e36431. doi: 10.1371/journal.pone.0036431 (PMC3349664; doi:10.1371/journal.pone.0036431)
Supplement: Table S1 — represents demographic information for different variables from BA9 region for the three different groups: Controls (N = 6), Schizophrenia (N = 6) and Bipolar Disorder (N = 6). This includes Age, Sex, Race, PMI (post mortem interval) in hours (hrs), Brain weight in grams (gms), pH, BISS (Bipolar Inventory of Signs and Symptoms Scale) and, MADRS (Montgomery Åsberg Depression Rating Scale). Total BISS score is cumulative of BISS1-5 (1 = depression, 2 = mania, 3 = irritability, 4 = anxiety, 5 = psychosis). * BISS and MADRS data was not available for one sample in each group. (DOCX) [file pone.0036431.s003.docx]

**Supplementary data- Tables**

**Table S1**. Sample Demographics for BA9.

|  | **Controls**  **(N=6)** | **Schizophrenia**  **(N=6)** | **Bipolar Disorder**  **(N=6)** |
| --- | --- | --- | --- |
| Age (Mean ± SD) | 54.5 ± 21.32 | 54.83 ± 4.79 | 47.6 ± 8.24 |
| Sex (Male : Female) | 6:0 | 4:2 | 3:3 |
| Race/Ethnicity  White Non-Hispanic  Hispanic | 5  1 | 4  2 | 5  1 |
| PMI, hrs. (Mean ± SD) | 24.15 ± 4.07 | 28.2 ± 4.76 | 29.08 ± 5.07 |
| Brain weight in gms  (Mean ± SD) | 1437.33±50.1 | 1298.33 ±141.97 | 1351.33±100.54 |
| pH (Mean ± SD) | 6.35 ± 0.36 | 6.27 ± 0.35 | 6.38 ± 0.33 |
| Total BISS score  (Mean ± SD) | 3.2 ± 3. 63* | 21.8 ± 11.49* | 43.4 ± 29.05* |
| BISS 1  (Mean ± SD) | 1.2 ± 2.68 | 5±3.46 | 12.2±11.52 |
| BISS 2  (Mean ± SD) | 1.6±2.61 | 6.2±5.07 | 19.8±14.27 |
| BISS 3  (Mean ± SD) | 0 | 1.8±1.31 | 5±4.42 |
| BISS 4  (Mean ± SD) | 0.4±0.89 | 4.4±4.98 | 5±4.69 |
| BISS 5  (Mean ± SD) | 0 | 3.2±3.35 | 1.4±1.67 |
| MADRS  (Mean ± SD) | 0* | 7.4±4.22* | 20.2±13.75* |

Abbreviations used: PMI, post mortem interval; BISS, Bipolar Inventory of Signs and Symptoms Scale ; MADRS Montgomery Åsberg Depression Rating Scale .Total BISS score is cumulative of BISS1-5 ( 1 = depression, 2 = mania, 3 = irritability, 4 = anxiety, 5 = psychosis). * BISS and MADRS data was not available for one sample in each group.
