# Supplementary material for: Differential Neuregulin 1 Cleavage in the Prefrontal Cortex and Hippocampus in Schizophrenia and Bipolar Disorder: Preliminary Findings
Source: PLoS One. 2012 May 10;7(5):e36431. doi: 10.1371/journal.pone.0036431 (PMC3349664; doi:10.1371/journal.pone.0036431)
Supplement: Table S2 — represents demographic information for different variables from hippocampal region for the three different groups: Controls (N = 5), Schizophrenia (N = 6) and Bipolar Disorder (N = 6). This includes Age, Sex, Race, PMI (post mortem interval) in hours (hrs), Brain weight in grams (gms), pH, BISS (Bipolar Inventory of Signs and Symptoms Scale) and, MADRS (Montgomery Åsberg Depression Rating Scale). Total BISS score is cumulative of BISS1-5 (1 = depression, 2 = mania, 3 = irritability, 4 = anxiety, 5 = psychosis). * BISS data was not available for one sample from the control group. ** shows the bipolar cohort to be significantly different in age from the control cohort (p = 0.004). (DOCX) [file pone.0036431.s004.docx]

**Table S2.** Sample Demographics for hippocampal region

|  | **Control**  **(N=5)** | **Schizophrenia (N=6)** | **Bipolar Disorder**  **(N=6)** |
| --- | --- | --- | --- |
| Age (Mean ± SD) | 64.2 ± 10.03 | 55.16 ± 4.71 | 48 ± 7.95 ** |
| Sex (Male : Female) | 4:1 | 4:2 | 3:3 |
| Race/Ethnicity  White Non-Hispanic  Hispanic | 5  0 | 4  2 | 6  0 |
| PMI, hrs. (Mean ± SD) | 23.68 ± 3.73 | 29.23 ± 4.3 | 30.08 ± 5.61 |
| Brain weight in gms (Mean ± SD) | 1389.2±101.95 | 1288.3±157.47 | 1345.5±98.15 |
| pH (Mean ± SD) | 6.29 ± 0.27 | 6.38 ± 0.13 | 6.34 ± 0.27 |
| Total BISS score  (Mean ± SD) | 2 ± 4* | 32.5 ± 28.15 | 40.16 ± 27.16 |
| Total BISS 1  (Mean ± SD) | 0 | 7±5.79 | 10.66±10.96 |
| Total BISS 2  (Mean ± SD) | 1.5 ±3 | 9.3±8.9 | 18.5±13.16 |
| Total BISS 3  (Mean ± SD) | 0 | 3.5±4.32 | 4.6±4.03 |
| Total BISS 4  (Mean ± SD) | 0.5±1 | 5.3±5.0 | 5±4.19 |
| Total BISS 5  (Mean ± SD) | 0 | 4.6±4.67 | 1.3±1.51 |
| MADRS  (Mean ± SD) | 0* | 12.5±13.05 | 18.16±13.27 |

Abbreviations used: PMI, post mortem interval; BISS, Bipolar Inventory of Signs and Symptoms Scale ; MADRS Montgomery Åsberg Depression Rating Scale .Total BISS score is cumulative of BISS1-5 ( 1 = depression, 2 = mania, 3 = irritability, 4 = anxiety, 5 = psychosis). * BISS data was not available for one sample from the control group. ** shows the bipolar cohort to be significantly different in age from the control cohort (p=0.004)
