# Supplementary material for: Differential Neuregulin 1 Cleavage in the Prefrontal Cortex and Hippocampus in Schizophrenia and Bipolar Disorder: Preliminary Findings
Source: PLoS One. 2012 May 10;7(5):e36431. doi: 10.1371/journal.pone.0036431 (PMC3349664; doi:10.1371/journal.pone.0036431)
Supplement: Table S3 — represents medication data for different variables from all the subjects used for our study. Variables shown include sample ID, Group (Control, schizophrenia (SCZ) or bipolar disorder (BPD)); Tissue availability (Brodmann’s area 9 (BA9) and/or hippocampus) and the different classes of medications that the subjects were on, including Antidepressant, Benzodiazapine, Neuroleptics and Mood stabilizer denoted by Y-Yes or N-No. (DOCX) [file pone.0036431.s005.docx]

**Table S3.** Sample medication data

| **ID** | **Group** | **BA9** | **Hippocampus** | **Antidepressant** | **Benzodiazapine** | **Neuroleptics** | **Mood stabilizer** |
| --- | --- | --- | --- | --- | --- | --- | --- |
| 33 | Control | Y | Y | N | N | N | N |
| 79 | Control | Y | Y | N | N | N | N |
| 83 | Control | Y | Y | N | N | N | N |
| 108 | Control | Y | N | N | N | N | N |
| 122 | Control | Y | N | N | N | N | N |
| 130 | Control | Y | Y | N | N | N | N |
| 162 | Control | N | Y | N | N | N | N |
| 53 | SCZ | Y | N | N | N | N | N |
| 62 | SCZ | Y | Y | N | Y | N | N |
| 84 | SCZ | Y | Y | N | N | N | N |
| 97 | SCZ | Y | Y | Y | N | Y | N |
| 106 | SCZ | Y | Y | Y | Y | Y | Y |
| 107 | SCZ | Y | Y | Y | Y | N | Y |
| 152 | SCZ | Y | Y | N | N | Y | N |
| 37 | BPD | Y | N | Y | N | N | N |
| 65 | BPD | Y | Y | N | N | N | N |
| 67 | BPD | Y | Y | N | N | N | N |
| 78 | BPD | Y | Y | Y | N | N | N |
| 91 | BPD | N | Y | Y | Y | N | Y |
| 96 | BPD | Y | Y | Y | N | N | N |
| 114 | BPD | Y | Y | Y | N | N | N |

Abbreviations used: SCZ, schizophrenia; BPD, bipolar disorder. Tissue availability and use of each class of medication for individual samples is indicated by Y-yes; N-No.
